# Calibration and evaluation of the relative biological effectiveness for carbon-ion radiotherapy in a new relative to a clinically applied treatment planning system

**DOI:** 10.1186/s13014-022-02181-5

**Published:** 2022-12-31

**Authors:** Weiwei Wang, Zhijie Huang, Wei Sun, Xufei Wang, Jingfang Zhao, Hao Shen

**Affiliations:** 1 Institute of Modern Physics, Applied Ion Beam Physics Laboratory, Fudan University, Shanghai, 200433 China; 2 Department of Medical Physics, Shanghai Proton and Heavy Ion Center, Fudan University Cancer Hospital, Shanghai Key Laboratory of Radiation Oncology (20dz2261000), Shanghai Engineering Research Center of Proton and Heavy Ion Radiation Therapy, 4365 Kangxin Road, Pudong District, Shanghai, 201315 China; 3 Department of Medical Physics, Shanghai Proton and Heavy Ion Center, Fudan University Cancer Hospital, 270 Dongan Road, Xuhui District, Shanghai, 200032 China

**Keywords:** RayStation, Syngo, RBE, LEM evaluation

## Abstract

**Background:**

The study objective was to validate the relative biological effectiveness (RBE) in RayStation for carbon-ion radiotherapy (CIRT) using the Syngo treatment planning system as reference.

**Methods:**

Local effect model I was established in RayStation (Ray-LEM) with the same parameters as in LEM I in Syngo (Syngo-LEM). Three cube plans covering most of the tumors treated at our center were generated with Syngo-LEM. Ray-LEM re-calculated the Syngo plans and compared the RBEs to the Syngo counterparts. The results showed that RayStation RBE was smaller than Syngo RBE. To ensure that Ray-LEM reproduced Syngo RBE, the observed deviations were used to scale the maximum RBE (RBEmax) in Ray-LEM. After this calibration, we further compared the RayStation RBE to Syngo RBE using additional plans in both homogeneous phantoms and patients, to ensure that the calibrated Ray-LEM reproduced Syngo RBE even with more complex planning features.

**Results:**

The calibration increased the RBEmax by 2.3% to raise the Ray-LEM RBE. The target mean RBE deviations in the phantom evaluation plans were median: 0.0 (minimum: − 1.1 to maximum: 0.7) %, and the target mean RBE deviations of the clinical target volumes of 16 patient cases were − 0.4 (− 1.5 to 0.2) %.

**Conclusions:**

The residual RBE difference between RayStation and Syngo was found to be ≤ 1.0%. Thus, we can propose to use RayStation for clinical CIRT treatment planning. However, the potential differences due to the absorbed beam model warrants further exploration.

**Supplementary Information:**

The online version contains supplementary material available at 10.1186/s13014-022-02181-5.

## Backgroud

Clinical carbon-ion radiotherapy (CIRT) originated from Japan in 1994 and has been shown to have physical and biological advantages over proton or photon radiotherapy [1]. Following the pioneers’ steps, our center established CIRT in 2015 with the Syngo (V13C, Siemens, Germany) treatment planning system (TPS). Unlike the constant relative biological effectiveness (RBE) of 1.1 used for proton radiotherapy [2], Syngo integrates the local effect model I (LEM I) [3] and a pencil beam algorithm (PBA) to provide RBE-weighted doses for clinical CIRT treatment planning. LEM I was designed to calculate a variable RBE value of carbon-ion in combination with the active scanning technique according to the local particle spectra [4]. Although several versions of LEMs have been developed [5–7], LEM I is the only LEM applied in a clinical TPS (LEM is hereafter referred to as LEM I). So far, our center has treated more than 5000 patients using LEM implemented in Syngo (Syngo-LEM) [8]. Preliminary results supported the effectiveness of CIRT with LEM [9–15]. However, the performance of Syngo has gradually declined with increasing clinical demands for CIRT. In 2019, our center deployed RayStation (V10B, RaySearch, Sweden) in the CIRT system as an alternative to Syngo TPS. Although RayStation is suggested to provide much faster and more robust treatment planning using the same LEM (Ray-LEM) and PBA for CIRT for clinical implementation, the RBE-weighted dose calculation in RayStation warrants comprehensive evaluation.

For CIRT planning, RBE-weighted and corresponding absorbed dose distributions given by RayStation should be consistent with Syngo. However, our preliminary evaluation showed that RayStation gave slightly smaller RBEs than Syngo. Therefore, we needed to examine Ray-LEM and the corresponding fragment spectra referring to Syngo. Inaniwa et al. [16] performed a similar study using a modified microdosimetric kinetic model (MKM). They matched the dose distributions from two TPSs based on a cube plan. Inspired by their methodology, we first compared the RBE distributions between RayStation and Syngo. Then, based on the difference, we determined a calibration factor and applied it to Ray-LEM. As a result, RayStation could reproduce the RBE of the Syngo TPS.

## Methods

### Local effect model (LEM)

LEM assumes that the biological effect of ionizing radiation on a cellular scale only depends on the mean number of killing events per cell, which is given by the total local energy deposition, no matter whether the energy has been deposited by photons or ions [17]. The local dose distribution can be simulated by the track structure model, while the averaged biological effect can be simulated using the Monte-Carlo method to account for its stochastic nature [4]. Syngo-LEM follows the same concepts while simplifying the processes for better efficiency. In CIRT planning, the initial slopes of the cell-survival curves of chordoma, given by the parameter $\alpha_{T, E}$ averaged over all ion types T and energies E, were pre-calculated and tabulated as RBEmax, i.e., the ratios of $\alpha_{T, E}$ values to the $\alpha_{X}$ value of photon radiation. As LEM was first applied in the treatment of skull-base tumors, $\left( {{\upalpha }/{\upbeta }} \right)_{X}$ was set at 2 Gy to account for late reactions of the central nervous system [18]. Although some centers set a different value for the certain tumors [19], our center used the same $\left( {{\upalpha }/{\upbeta }} \right)_{X}$ of 2 Gy for both tumors and normal tissues. In mixed carbon-ion irradiations, Syngo-LEM calculates the dose-weighted $\alpha_{T, E}$ using the table based on corresponding fragment spectra [20] and determines the final RBE through a Monte-Carlo simulation.

### Configuration of Ray-LEM

All model parameters in Ray-LEM were set to the same values as in Syngo-LEM. The $\alpha_{T, E}$ table in Syngo-LEM was converted to RBEmax-values and imported into Ray-LEM [21]. The fragment spectra in Syngo were processed by RaySearch to derive a file compatible with RayStation as consistent as possible. However, the remaining differences were that the clinically used number of Monte-Carlo samples was 500 in Syngo but 1000 in RayStation. In addition, the step size in-depth in the spectra was changed from a dose gradient-dependent step size in Syngo to a fixed value of 0.1 cm in RayStation, and the energy resolution was also different for different ion types. Because Ray-LEM was presumed to be consistent with Syngo-LEM, the aforementioned parameters in Syngo-LEM were used in Ray-LEM, to minimize the potential difference from Syngo-LEM and to clarify the origin.

### Calibration of Ray-LEM using Syngo-LEM as reference

**Fig. 1:**
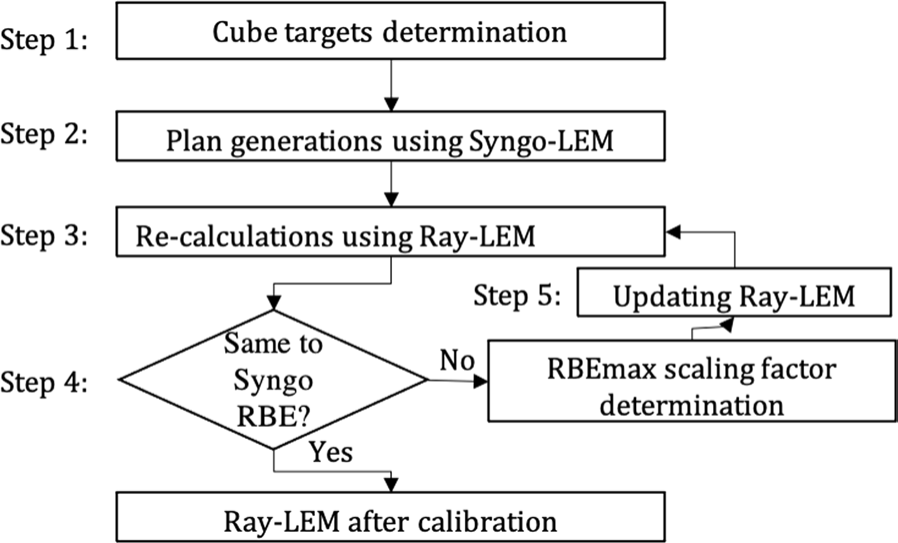
The flowchart of the calibration of Ray-LEM

The flowchart of the calibration shows in Fig. 1. In step 1, we defined the cube targets for calibration. Following the procedure in Inaniwa et al. [16], we generated three 6.0 × 6.0 × 6.0 cm^3^ cube targets in a WP. The depths of the center of the target volumes were 4.0, 10.0, and 16.0 cm, respectively. Depths and sizes of the target volumes aimed to cover most of the tumors treated at our center.

In step 2, based on the cube targets, we generated three single beam treatment plans in Syngo. The RBE-weighted prescription was 4.0 Gy (RBE) with LEM. The dose grid size was 0.3 cm.

In step 3, Syngo-optimized plans were imported into RayStation, where dose re-calculations were performed based on Ray-LEM. By recalculation of the treatment plan with RayStation based on the fluence distribution, we obtained a new RBE-weighted and new absorbed dose distribution. As only the ratio of both reflects the RBE distribution, the RBE-weighted dose distribution of RayStation was normalized to that of Syngo. This implies that the absorbed dose distribution of RayStation was scaled as well. In this way, the RBE distribution is conserved and the deviations in RBE could now be read directly from the deviations in absorbed doses. Step 4 compared RayStation RBE to that of Syngo RBE. If the absorbed doses of RayStation were not the same as those of Syngo, we went to step 5. In this step, the average deviation of the absorbed doses was applied as a correction to the RBE distribution by applying it to the RBEmax-values of the input database for all depths and ion types. Steps 3–5 were iteratively performed until the averaged RayStation RBE was the same as in Syngo RBE. In the following, this procedure is termed as calibration of Ray-LEM.

This calibration procedure was chosen since we cannot access the RBE (and its deviation) in the TPS directly and to obtain the RBE deviation from the absorbed dose deviations, identical RBE-weighted doses are required.

### Validation of Ray-LEM after calibration

The above calibration was derived using three simple geometric fields. However, real treatment plans may differ in field number, target shapes, prescriptions, and depths. Therefore, a variety of additional treatment plans differing in these features were re-calculated with RayStation after being generated with the Syngo. Similarly, the RBE-weighted dose of RayStation was normalized to that of Syngo, and the absorbed dose was scaled accordingly (to access RBE deviations). Residual deviations in absorbed dose were then used to characterize residual deviations in RBE and thus the accuracy of the calibration for the general cases. A similar approach was followed by Inaniwa et al. [16] and Fossati et al. [22]. Table 1 lists the details of the plan parameters.

**Table 1:** Parameters of the evaluation plans in WPs

Iso^a^	Target shape		Target size^b^	Prescription^c^				Beam arrangements^d^			Dose grid	
	Cube	Sphere		3.0	3.5	4.0	4.5	Single	Orthogonal	Opposed	2 mm	3 mm
3	√	√	1.0–5.0			√		√	√		√	
7	√		4.0–12.0	√	√	√	√	√				√
	√	√	4.0–12.0			√		√	√	√		√
11	√	√	4.0–12.0			√		√	√	√		√
15	√	√	4.0–12.0			√		√	√	√		√

^a^Distances from the surface of WPs to the target centers

^b^Dimensions of the cube targets or the diameters of the sphere targets (cm), the step size is 2.0 cm

^c^Prescribed RBE-weighted doses [Gy (RBE)] in Syngo

^d^Beam arrangements are the single beam, two orthogonal beams, and two opposed beams

In addition, we randomly selected 16 patients with typical target volumes and dose prescriptions used at our center. These included eight head and neck cases (two eye cancers, two parotid cancers, two nasopharynx carcinomas (NPCs), and two lacrimal cancers), two non-small-cell-lung-cancer cases, four abdominal cases (two liver cancers and two pancreatic cancers), and two prostate cases. Their CIRT treatment plans from Syngo were also re-calculated based on the same CT image sets and the corresponding stopping power conversion curve. After calibration, the mean RBEs of the clinical target volumes (CTVs) in RayStation were compared with those in Syngo in the same way as for the cube plans.

## Results

### Calibration

**Fig. 2:**
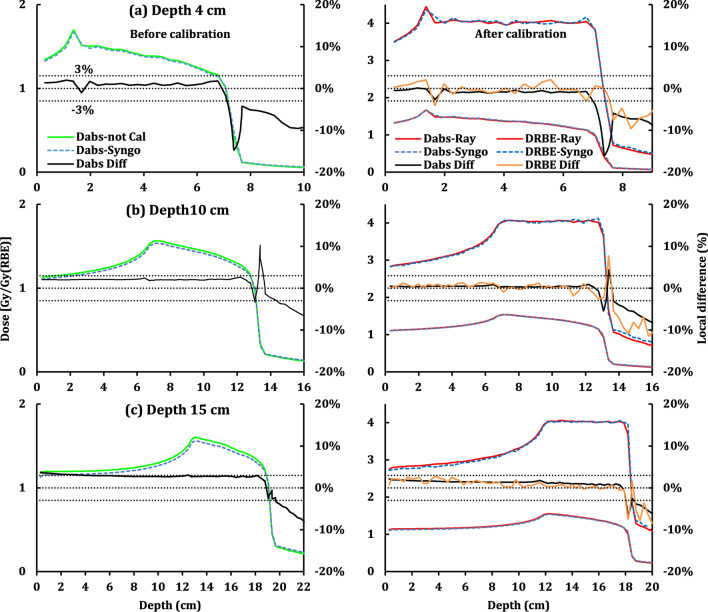
RBE-weighted (D_RBE_-Ray) and absorbed (D_abs_-Ray) DDDs at the central axis of the calibration plans after calibrations and the corresponding RBE-weighed (D_RBE_-Syngo) and absorbed (D_abs_-Syngo) DDDs from Syngo. Local differences are also displayed in the figures. Absorbed DDDs before calibration (D_abs-not Cal_) in the left panel are shown for comparisons. The centers of targets of (**a**–**c**) are 4.0, 10.0, and 15.0 cm from the surface of WPs, respectively

Figure 2 shows the absorbed and RBE-weighted depth dose distributions (DDDs) of both TPS after normalization of the RBE-weighted doses as well as the corresponding local differences on the central axis of three calibration plans. The figures in the left panel are the Ray-LEM results before calibration while the figures in the right panel are the Ray-LEM results after calibration. Table 2 shows the corresponding target mean doses. The correction by the RBEmax calibration factor was + 2.3%. The dose distributions after calibrations are in good agreement with those from Syngo plans. Although there are some fluctuations, the local difference of both RBE-weighted and absorbed doses agree well, which indicates the remaining RBE difference is small. The largest difference was found for the plan centered at 4.0 cm (Fig. 2a) from the surface of WP, where the target mean absorbed doses after calibration is still 0.01 Gy lower than that from Syngo.

**Table 2:** Target mean RBE-weighted and absorbed doses of the three calibration plans

Calibration^a^	Depth 4 cm			Depth 10 cm			Depth 16 cm		
	RS RC^b^	RS Ca^c^	Syn^d^	RS RC	RS Ca	Syn	RS RC	RS Ca	Syn
Dabs [Gy]	1.41	1.38	1.39	1.41	1.39	1.39	1.44	1.41	1.41
DRBE [Gy (RBE)]	4.06			4.03			4.03		

^a^They have cubic targets with a size of 6.0 × 6.0 × 6.0 cm^3^; the prescriptions are 4.0 Gy (RBE)

^b^The re-calculated doses before calibrations from RayStation based on the Syngo plans

^c^The re-calculated doses after calibrations from RayStation based on the Syngo plans

^d^Doses from the Syngo optimized plans

### Phantom evaluation

**Fig. 3:**
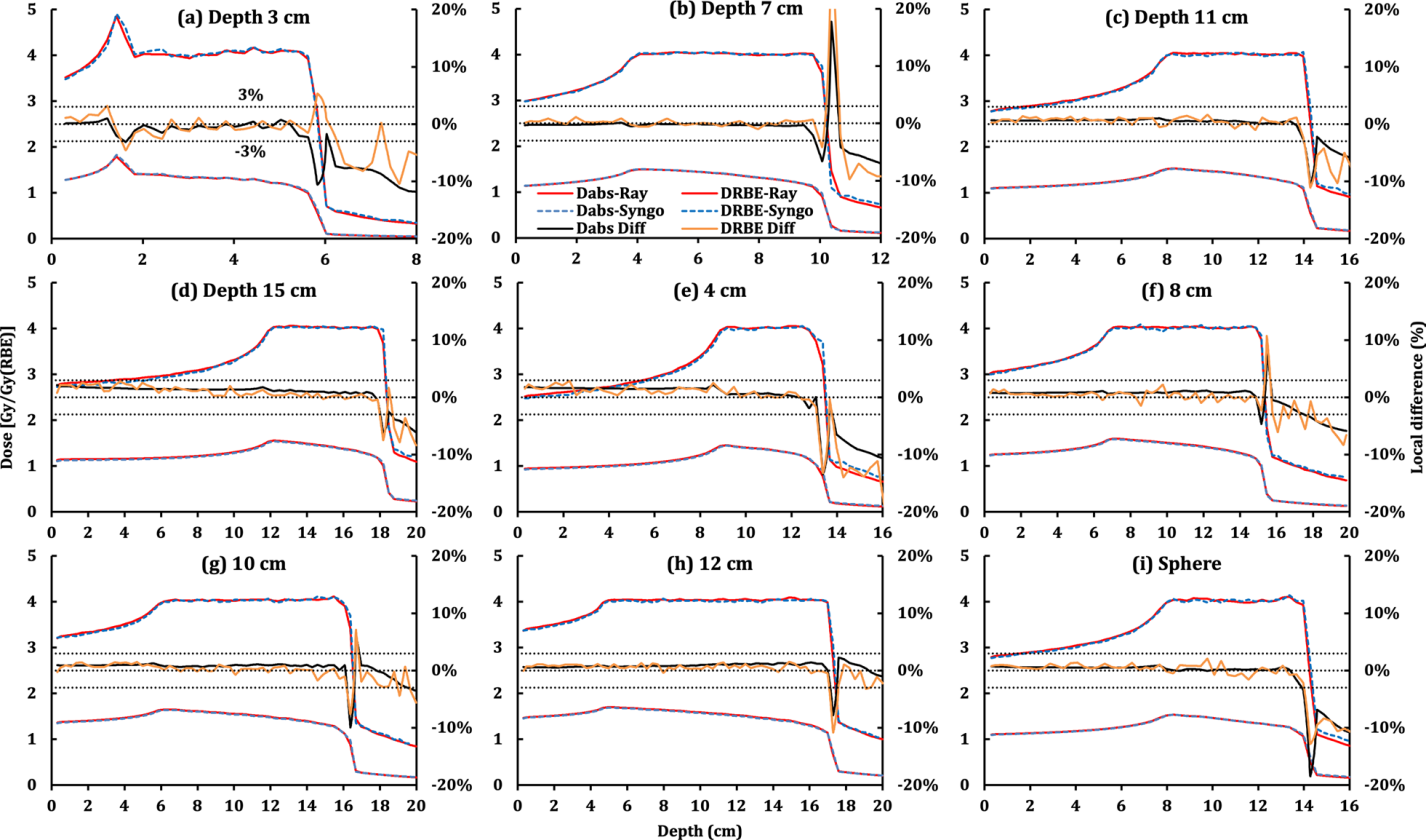
Comparison of RBE-weighted (D_RBE_-Ray) and absorbed (D_abs_-Ray) doses of the recalculated plans from Raystation and the corresponding RBE-weighted (D_RBE_-Syngo) and absorbed (D_abs_-Syngo) doses from Syngo, together with the local differences. The prescription doses of (**a**–**d**) are 3.0, 3.5, 4.0, and 4.5 Gy (RBE) respectively. The beam arrangements of (**c**, **e**, and **f**) are a single beam, two orthogonal beams, and two opposed beams

**Fig. 4:**
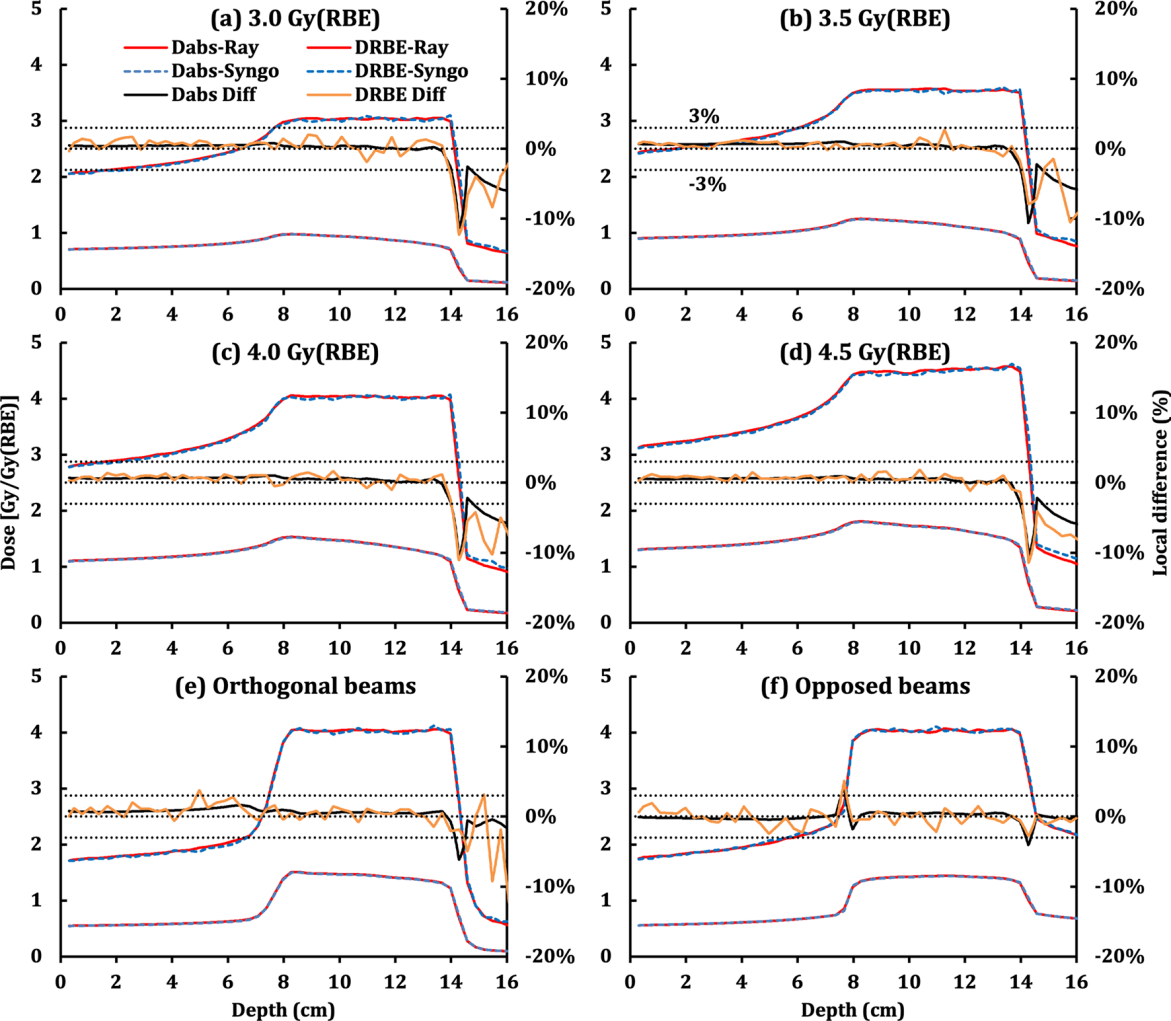
Comparison of RBE-weighted (D_RBE_-Ray) and absorbed (D_abs_-Ray) doses of the re-calculated plans from RayStation and the corresponding RBE-weighted (D_RBE_-Syngo) and absorbed (D_abs_-Syngo) doses from Syngo. Local differences are also displayed in the figures. Among them, the centers of targets of (**a**–**d**) are 3.0, 7.0, 11.0, and 15.0 cm from the surface of WPs, respectively. Target sizes of (**e**, **c**, **f**–**h**) are 4.0, 6.0, 8.0, 10.0, and 12.0 cm, respectively. The target shapes of (**c** and **i**) refer to cubic and spherical target volumes, respectively

After RBE calibration was applied and the RBE-weighted dose normalization was performed, the RBE difference of the target mean RBE between RayStation and Syngo was in median: 0.0 (minimum: − 1.1 to maximum: 0.7) %. Detailed results are summarized in Additional file 1: Table S1. Figures 3 and 4 display the results of the plans whose targets were centered at 11.0 cm depth. Furthermore, Fig. 3 shows the comparisons of different target geometries, while Fig. 4 evaluates different prescriptions and beam arrangements.

Figure 3a–d show the absorbed and RBE-weighted DDDs as well as the corresponding local differences on the central axis of four cube plans. Their targets centered at the depths of 3.0 cm (a), 7.0 cm (b), 11.0 cm (c), and 15.0 cm (d), respectively. In Fig. 3a, the proximal of DDDs have a spike, which may be considered clinically by using a range shifter. At 11 cm depth, the cube sizes of Fig. 3e, c, f–h vary from 4.0 cm (e), 6.0 cm (c), 8.0 cm (f), and 10.0 cm (g) to 12.0 cm (h). In addition, Fig. 3i shows the result for a spherical target volume.

Figures 4a–d show the absorbed and RBE-weighted DDDs as well as the corresponding local differences at the central axis of the cube plans. Their prescriptions were 3.0 (a), 3.5 (b), 4.0 (c), and 4.5 Gy (RBE) (d), where Fig. 4c is the same as Fig. 3c while other cube plans use new prescriptions. Figure 4c, e, f show the DDDs together with the corresponding local differences at the center axis of the cube plans with a single beam (c), two orthogonal beams (e), and two opposed beams (f).

### Evaluation of patients’ plans

Similarly, the comparison of patient cases was also conducted based on the calibrated Ray-LEM and the RBE-weighted doses were again scaled to match those of Syngo. The averaged/median clinical RBE-weighted prescriptions per fraction of 16 patients were 3.5 (2.75–9.0) Gy(RBE). The mean RBE deviations of CTVs between RayStation and that of Syngo were − 0.4 (− 1.5 to 0.2) %. Table 3 gives detailed information.

**Table 3:** Comparison of Ray-LEM and Syngo-LEM in 16 patient cases

List	Site	Fraction	D_RBE/_fx [ Gy(RBE)]	RBE Deviation (%)
P1	Eye	5	9.0	− 1.2
P2	Eye	5	9.0	− 1.5
P3	Parotid	20	3.5	0.2
P4	Parotid	20	3.5	0.2
P5	NPC	5	3.5	0.2
P6	NPC	5	3.5	0.0
P7	Lacrimal	18	3.5	0.0
P8	Lacrimal	18	3.5	− 0.2
P9	Lung	10	6.5	− 0.3
P10	Lung	8	8.0	− 0.5
P11	Liver	10	2.75	0.0
P12	Liver	10	6.0	− 0.5
P13	Pancreas	18	3.0	− 0.4
P14	Pancreas	18	3.0	− 0.4
P15	Prostate	12	3.8	− 1.0
P16	Prostate	12	3.8	− 1.20

## Discussion

In this study, Ray-LEM was established with the same parameters and the same fragment and RBEmax input data were used for Ray-LEM and Syngo-LEM. Three cube plans were generated to quantify the potential deviations and to derive a scaling factor to correct the RBE of Ray-LEM to that of Syngo. After that, we applied the treatment plans within the WPs of different conditions and patient cases to evaluate the calibrated Ray-LEM. The results showed that the remaining difference was minimal.

Syngo was utilized to treat over 5000 patients in our center and proved to be effective in various diseases [9–15]. To make the former experience also valid for the future CIRT practice, we choose to calibrate the RBE of RayStation to that of Syngo, instead of scaling the current dose prescriptions. In this way, we can achieve the delivery of the same RBEs in both TPS. Furthermore, we also performed analysis on patients with different tumors and sites. Based on the comprehensive evaluation, we could adopt RayStation for the clinical CIRT planning and the same clinical results are expected as for Syngo TPS.

Without calibration, the local difference curves of three calibration cubes implied that RayStation underestimates the RBE. As the underestimation in the beam entrances and target regions was uniform, we implemented a single calibration factor. Furthermore, our ongoing analysis showed that the linear energy (LET) of the fragment spectra in RayStation was overestimated. While carbon-ions are the main contributor to the dose averaged LET (LETd) and thus to the RBE, the ion types from Z = 1–5 also contribute to the LETd in the distal part of the SOBP. Our approach could therefore not mitigate the RBE deviation in this region.

The largest difference after calibration was found in the tail behind the SOBP. Three factors likely played a role there. The main reason was the absorbed dose difference in this region. Our unpublished data showed that absorbed doses from RayStation were closer to the measurements than for Syngo. Second, the rebinned spectra by RayStation were not exactly the same as those in Syngo, which caused additional RBE differences. Third, the number of random sampling in Syngo-LEM was 500, which was a compromise between statistical accuracy and optimization speed. Therefore, the calculated RBE-weighted doses would have over 3% uncertainty [4]. Because the calculation speed of RayStation improved, the number of simulated events was set to 1000, which especially improved the accuracy in the tail behind the SOBP.

The present study generated calibration and evaluation plans in Syngo. RayStation only re-calculated the dose distribution instead of re-optimizing the dose distribution to avoid the absorbed dose differences due to different optimizers. After calibration, the evaluation plans whose targets centered at 11 cm from the surface of WPs in RayStation were re-optimized based on the same RBE-weighted doses. The target mean absorbed dose deviation to the Syngo-optimized plans was − 0.7% (− 2.8 to 7.1%). The detailed results are provided Additional file 1: Table S1. These results indicate that optimization may lead to different absorbed dose distributions based on the same RBE-weighted doses.

Our study only involved the fragment spectra of our facility. It is noteworthy that the beam quality of beamlines in other facilities may yield different fragment spectra. This may cause some RBE differences even when based on the same LEM. However, we investigated various conditions corresponding to very different fragment spectra, so it seems likely that the results would be similar. Only a few patients were evolved for evaluation in the present study. Hence, further evaluations using more patients with different tumors are warranted.

## Conclusions

In this study, we first established Ray-LEM using the same parameters and the same fragment and RBEmax input data as the clinical applied Syngo-LEM. The comparison to Syngo RBE showed that Ray-LEM underestimated RBE. This difference was corrected by increasing the RBEmax in Ray-LEM by 2.3% to that of Syngo. After calibration, we comprehensively evaluated Ray-LEM referring to Syngo-LEM by using both phantom plans and patient cases. The results showed that the residual RBE difference between the two TPSs was ≤ 1.0%. Based on this result, it is justified to adopt the Ray-LEM for future clinical CIRT and similar clinical results can be expected as for the Syngo TPS.

## Supplementary Information

**Additional file 1**. **Table S1**: All the treatment plans used for the evaluation of the Ray-LEM.

## Data Availability

The datasets used and analyzed during the current study are available from the corresponding author on reasonable request.

## Abbreviations

CIRT: Carbon-ion radiotherapy
Ray-LEM: Local effect model I in RayStation
Syngo-LEM: LEM I in Syngo
WP: Water phantom
RBEmax: The maximum value of relative biological effect
TPS: Treatment planning system
LEM I: Local effect model I
PBA: Pencil beam algorithm
MKM: Modified microdosimetric kinetic model
CTV: Clinical target volume
DDD: Depth dose distribution
D_RBE_-Ray: RBE-weighted doses in RayStation
D_abs_-Ray: Absorbed doses in RayStation
D_RBE_-Syngo: RBE-weighted doses in Syngo
D_abs_-Syngo: Absorbed doses in Syngo
LET: Linear energy transfer
LETd: Dose averaged LET
