# Supplementary material for: Calibration and evaluation of the relative biological effectiveness for carbon-ion radiotherapy in a new relative to a clinically applied treatment planning system
Source: Radiat Oncol. 2022 Dec 31;17:219. doi: 10.1186/s13014-022-02181-5 (PMC9805684; doi:10.1186/s13014-022-02181-5)
Supplement: Supplementary file 1 — Additional file 1. Table S1: All the treatment plans used for the evaluation of the Ray-LEM. [file 13014_2022_2181_MOESM1_ESM.docx]

**Supplementary material**

**Table S1** All the treatment plans used for the evaluation of the Ray-LEM

| ^1^ISO (cm) | Target shape | ^2^Target size (cm) | Dpre [Gy(RBE)] | Beam  arrangement | DRBE [Gy(RBE)] | Dabs (Gy) | | | RBE |  | |
| --- | --- | --- | --- | --- | --- | --- | --- | --- | --- | --- | --- |
|  |  |  |  |  | Syngo | ^3^Ray-RF | Syngo | Devation | | | ^4^Ray-RO |
| 3 | cube | 5 | 4 | Single | 4.13 | 1.39 | 1.39 | 0.0% | | | - |
| 3 | cube | 3 | 4 | Single | 4.07 | 1.30 | 1.29 | -0.8% | | | - |
| 3 | cube | 1 | 4 | Single | 4.24 | 1.29 | 1.28 | -0.8% | | | - |
| 3 | cube | 5 | 4 | Orthogonal | 4.03 | 1.38 | 1.38 | 0.0% | | | - |
| 3 | cube | 3 | 4 | Orthogonal | 4.02 | 1.31 | 1.30 | -0.8% | | | - |
| 3 | cube | 1 | 4 | Orthogonal | 4.02 | 1.19 | 1.18 | -0.8% | | | - |
| 3 | Sphere | 5 | 4 | Single | 4.08 | 1.34 | 1.34 | 0.0% | | | - |
| 3 | Sphere | 3 | 4 | Single | 4.08 | 1.28 | 1.27 | -0.8% | | | - |
| 3 | Sphere | 1 | 4 | Single | 4.11 | 1.21 | 1.20 | -0.8% | | | - |
| 3 | Sphere | 5 | 4 | Orthogonal | 4.04 | 1.32 | 1.32 | 0.0% | | | - |
| 3 | Sphere | 3 | 4 | Orthogonal | 4.04 | 1.26 | 1.25 | -0.8% | | | - |
| 3 | Sphere | 1 | 4 | Orthogonal | 4.03 | 1.15 | 1.15 | 0.0% | | | - |
| 7 | cube | 12 | 4 | Single | 3.98 | 1.48 | 1.48 | 0.0% | | | - |
| 7 | cube | 10 | 4 | Single | 4.03 | 1.48 | 1.48 | 0.0% | | | - |
| 7 | cube | 8 | 4 | Single | 4.00 | 1.42 | 1.43 | 0.7% | | | - |
| 7 | cube | 6 | 4 | Single | 4.02 | 1.36 | 1.37 | 0.7% | | | - |
| 7 | cube | 4 | 4 | Single | 4.04 | 1.33 | 1.33 | 0.0% | | | - |
| 7 | cube | 12 | 4 | Orthogonal | 4.02 | 1.53 | 1.53 | 0.0% | | | - |
| 7 | cube | 10 | 4 | Orthogonal | 4.02 | 1.49 | 1.49 | 0.0% | | | - |
| 7 | cube | 8 | 4 | Orthogonal | 4.03 | 1.46 | 1.45 | -0.7% | | | - |
| 7 | cube | 6 | 4 | Orthogonal | 4.03 | 1.40 | 1.41 | 0.7% | | | - |
| 7 | cube | 4 | 4 | Orthogonal | 4.01 | 1.33 | 1.33 | 0.0% | | | - |
| 7 | Sphere | 12 | 4 | Single | 3.98 | 1.42 | 1.42 | 0.0% | | | - |
| 7 | Sphere | 10 | 4 | Single | 4.04 | 1.43 | 1.43 | 0.0% | | | - |
| 7 | Sphere | 8 | 4 | Single | 4.04 | 1.39 | 1.39 | 0.0% | | | - |
| 7 | Sphere | 6 | 4 | Single | 4.05 | 1.35 | 1.36 | 0.7% | | | - |
| 7 | Sphere | 4 | 4 | Single | 4.05 | 1.30 | 1.30 | 0.0% | | | - |
| 7 | cube | 12 | 4 | Opposed | 4.02 | 1.54 | 1.53 | -0.6% | | | - |
| 7 | cube | 10 | 4 | Opposed | 4.02 | 1.50 | 1.50 | 0.0% | | | - |
| 7 | cube | 8 | 4 | Opposed | 4.02 | 1.45 | 1.45 | 0.0% | | | - |
| 7 | cube | 6 | 4 | Opposed | 4.02 | 1.40 | 1.40 | 0.0% | | | - |
| 7 | cube | 4 | 4 | Opposed | 4.03 | 1.34 | 1.34 | 0.0% | | | - |
| 7 | Sphere | 12 | 4 | Orthogonal | 4.03 | 1.47 | 1.47 | 0.0% | | | - |
| 7 | Sphere | 10 | 4 | Orthogonal | 4.03 | 1.44 | 1.44 | 0.0% | | | - |
| 7 | Sphere | 8 | 4 | Orthogonal | 4.03 | 1.41 | 1.40 | -0.7% | | | - |
| 7 | Sphere | 6 | 4 | Orthogonal | 4.03 | 1.35 | 1.36 | 0.7% | | | - |
| 7 | Sphere | 4 | 4 | Orthogonal | 4.04 | 1.30 | 1.30 | 0.0% | | | - |
| 7 | Sphere | 12 | 4 | Opposed | 4.02 | 1.54 | 1.53 | -0.6% | | | - |
| 7 | Sphere | 10 | 4 | Opposed | 4.02 | 1.50 | 1.50 | 0.0% | | | - |
| 7 | Sphere | 8 | 4 | Opposed | 4.02 | 1.45 | 1.45 | 0.0% | | | - |
| 7 | Sphere | 6 | 4 | Opposed | 4.02 | 1.40 | 1.40 | 0.0% | | | - |
| 7 | Sphere | 4 | 4 | Opposed | 4.03 | 1.34 | 1.34 | 0.0% | | | - |
| 11 | Cube | 12 | 3 | Single | 2.98 | 0.96 | 0.95 | -1.0% | | | 0.95 |
| 11 | Cube | 12 | 3.5 | Single | 3.47 | 1.21 | 1.21 | 0.0% | | | 1.20 |
| 11 | Cube | 12 | 4 | Single | 4.01 | 1.51 | 1.51 | -0.7% | | | 1.48 |
| 11 | Cube | 12 | 4.5 | Single | 4.47 | 1.80 | 1.79 | -0.6% | | | 1.78 |
| 11 | Cube | 10 | 3 | Single | 3.02 | 0.95 | 0.95 | 0.0% | | | 0.94 |
| 11 | Cube | 10 | 3.5 | Single | 3.52 | 1.21 | 1.21 | 0.0% | | | 1.20 |
| 11 | Cube | 10 | 4 | Single | 4.03 | 1.49 | 1.49 | 0.0% | | | 1.48 |
| 11 | Cube | 10 | 4.5 | Single | 4.53 | 1.80 | 1.80 | 0.0% | | | 1.78 |
| 11 | Cube | 8 | 3 | Single | 3.02 | 0.93 | 0.92 | -1.1% | | | 0.91 |
| 11 | Cube | 8 | 3.5 | Single | 3.52 | 1.18 | 1.17 | -0.8% | | | 1.15 |
| 11 | Cube | 8 | 4 | Single | 4.02 | 1.44 | 1.44 | -0.7% | | | 1.42 |
| 11 | Cube | 8 | 4.5 | Single | 4.52 | 1.75 | 1.75 | 0.0% | | | 1.72 |
| 11 | Cube | 6 | 3 | Single | 3.02 | 0.89 | 0.89 | 0.0% | | | 0.88 |
| 11 | Cube | 6 | 3.5 | Single | 3.53 | 1.13 | 1.13 | 0.0% | | | 1.12 |
| 11 | Cube | 6 | 4 | Single | 4.03 | 1.39 | 1.39 | 0.0% | | | 1.39 |
| 11 | Cube | 6 | 4.5 | Single | 4.53 | 1.69 | 1.69 | 0.0% | | | 1.67 |
| 11 | Cube | 4 | 3 | Single | 3.03 | 0.86 | 0.86 | 0.0% | | | 0.84 |
| 11 | Cube | 4 | 3.5 | Single | 3.52 | 1.08 | 1.08 | 0.0% | | | 1.07 |
| 11 | Cube | 4 | 4 | Single | 4.02 | 1.34 | 1.33 | 0.0% | | | 1.32 |
| 11 | Cube | 4 | 4.5 | Single | 4.53 | 1.62 | 1.62 | 0.0% | | | 1.60 |
| 11 | Cube | 12 | 4 | Orthogonal | 4.02 | 1.56 | 1.55 | -0.6% | | | 1.51 |
| 11 | Cube | 10 | 4 | Orthogonal | 4.02 | 1.52 | 1.51 | -0.7% | | | 1.47 |
| 11 | Cube | 8 | 4 | Orthogonal | 4.02 | 1.47 | 1.47 | 0.0% | | | 1.43 |
| 11 | Cube | 6 | 4 | Orthogonal | 4.02 | 1.41 | 1.41 | -0.7% | | | 1.38 |
| 11 | Cube | 4 | 4 | Orthogonal | 4.02 | 1.36 | 1.36 | 0.0% | | | 1.33 |
| 11 | Sphere | 12 | 4 | Orthogonal | 4.03 | 1.50 | 1.49 | -0.7% | | | 1.49 |
| 11 | Sphere | 10 | 4 | Orthogonal | 4.03 | 1.46 | 1.46 | 0.0% | | | 1.46 |
| 11 | Sphere | 8 | 4 | Orthogonal | 4.03 | 1.42 | 1.42 | 0.0% | | | 1.42 |
| 11 | Sphere | 6 | 4 | Orthogonal | 4.03 | 1.38 | 1.38 | 0.0% | | | 1.40 |
| 11 | Sphere | 4 | 4 | Orthogonal | 4.02 | 1.31 | 1.31 | 0.0% | | | 1.32 |
| 11 | Sphere | 12 | 4 | Single | 3.95 | 1.43 | 1.42 | -0.7% | | | 1.48 |
| 11 | Sphere | 10 | 4 | Single | 4.03 | 1.45 | 1.44 | -0.7% | | | 1.46 |
| 11 | Sphere | 8 | 4 | Single | 4.03 | 1.41 | 1.41 | 0.0% | | | 1.42 |
| 11 | Sphere | 6 | 4 | Single | 4.03 | 1.37 | 1.37 | 0.0% | | | 1.40 |
| 11 | Sphere | 4 | 4 | Single | 4.03 | 1.31 | 1.30 | -0.8% | | | 1.32 |
| 11 | Cube | 12 | 4 | Opposed | 4.02 | 1.56 | 1.55 | -0.6% | | | 1.51 |
| 11 | Cube | 10 | 4 | Opposed | 4.02 | 1.52 | 1.51 | -0.7% | | | 1.48 |
| 11 | Cube | 8 | 4 | Opposed | 4.02 | 1.47 | 1.47 | 0.0% | | | 1.43 |
| 11 | Cube | 6 | 4 | Opposed | 4.02 | 1.41 | 1.41 | 0.0% | | | 1.51 |
| 11 | Cube | 4 | 4 | Opposed | 4.02 | 1.35 | 1.35 | 0.0% | | | 1.34 |
| 11 | Sphere | 12 | 4 | Opposed | 4.03 | 1.50 | 1.49 | -0.7% | | | 1.49 |
| 11 | Sphere | 10 | 4 | Opposed | 4.03 | 1.47 | 1.46 | -0.7% | | | 1.48 |
| 11 | Sphere | 8 | 4 | Opposed | 4.03 | 1.46 | 1.46 | 0.0% | | | 1.42 |
| 11 | Sphere | 6 | 4 | Opposed | 4.03 | 1.38 | 1.38 | 0.0% | | | 1.38 |
| 11 | Sphere | 4 | 4 | Opposed | 4.03 | 1.31 | 1.31 | 0.0% | | | 1.33 |
| 15 | Cube | 4 | 4 | Single | 3.96 | 1.33 | 1.33 | 0.0% | | | - |
| 15 | Cube | 6 | 4 | Single | 4.01 | 1.41 | 1.40 | -0.7% | | | - |
| 15 | Cube | 8 | 4 | Single | 3.97 | 1.44 | 1.43 | -0.7% | | | - |
| 15 | Cube | 10 | 4 | Single | 4.08 | 1.55 | 1.54 | -0.6% | | | - |
| 15 | Cube | 12 | 4 | Single | 3.99 | 1.53 | 1.52 | -0.7% | | | - |
| 15 | Cube | 4 | 4 | Orthogonal | 4.04 | 1.40 | 1.40 | 0.0% | | | - |
| 15 | Cube | 6 | 4 | Orthogonal | 4.03 | 1.46 | 1.45 | -0.7% | | | - |
| 15 | Cube | 8 | 4 | Orthogonal | 4.03 | 1.51 | 1.50 | -0.7% | | | - |
| 15 | Cube | 10 | 4 | Orthogonal | 4.03 | 1.55 | 1.54 | -0.6% | | | - |
| 15 | Cube | 12 | 4 | Orthogonal | 4.03 | 1.59 | 1.58 | -0.6% | | | - |
| 15 | Cube | 4 | 4 | Opposed | 4.04 | 1.38 | 1.38 | 0.0% | | | - |
| 15 | Cube | 6 | 4 | Opposed | 4.03 | 1.45 | 1.44 | -0.7% | | | - |
| 15 | Cube | 8 | 4 | Opposed | 4.02 | 1.50 | 1.49 | -0.7% | | | - |
| 15 | Cube | 10 | 4 | Opposed | 4.03 | 1.55 | 1.54 | -0.6% | | | - |
| 15 | Cube | 12 | 4 | Opposed | 4.03 | 1.59 | 1.58 | -0.6% | | | - |
| 15 | Sphere | 4 | 4 | Single | 4.05 | 1.34 | 1.34 | 0.0% | | | - |
| 15 | Sphere | 6 | 4 | Single | 4.04 | 1.39 | 1.38 | -0.7% | | | - |
| 15 | Sphere | 8 | 4 | Single | 3.97 | 1.39 | 1.39 | 0.0% | | | - |
| 15 | Sphere | 10 | 4 | Single | 4.10 | 1.51 | 1.50 | -0.7% | | | - |
| 15 | Sphere | 12 | 4 | Single | 3.99 | 1.47 | 1.46 | -0.7% | | | - |
| 15 | Sphere | 4 | 4 | Orthogonal | 4.11 | 1.37 | 1.37 | 0.0% | | | - |
| 15 | Sphere | 6 | 4 | Orthogonal | 4.11 | 1.43 | 1.42 | -0.7% | | | - |
| 15 | Sphere | 8 | 4 | Orthogonal | 4.11 | 1.47 | 1.47 | 0.0% | | | - |
| 15 | Sphere | 10 | 4 | Orthogonal | 4.03 | 1.49 | 1.48 | -0.7% | | | - |
| 15 | Sphere | 12 | 4 | Orthogonal | 4.14 | 1.56 | 1.55 | -0.6% | | | - |
| 15 | Sphere | 4 | 4 | Opposed | 4.10 | 1.36 | 1.36 | 0.0% | | | - |
| 15 | Sphere | 6 | 4 | Opposed | 4.11 | 1.43 | 1.42 | -0.7% | | | - |
| 15 | Sphere | 8 | 4 | Opposed | 4.12 | 1.48 | 1.47 | -0.7% | | | - |
| 15 | Sphere | 10 | 4 | Opposed | 4.03 | 1.49 | 1.48 | -0.7% | | | - |
| 15 | Sphere | 12 | 4 | Opposed | 4.12 | 1.55 | 1.54 | -0.6% | | | - |

^1^The distances to the target centers from the surface of WPs

^2^The dimensions of the cube targets or the diameters of the sphere targets

^3^The re-calculated target mean absorbed doses by Ray-LEM

^4^The re-optimized target mean absorbed doses by Ray-LEM
